# PARTNERS2: a protocol for the development of a core outcome set for use in mental health trials involving people with schizophrenia or bipolar disorder in a community setting

**DOI:** 10.1186/1745-6215-16-S1-P28

**Published:** 2015-05-29

**Authors:** Thomas Keeley, Humera Khan, Vanessa Pinfold, Paula Williamson, Jonathan Mathers, Linda Davies, Ruth Sayers, Elizabeth England, Siobhan Reilly, Richard Byng, Linda Gask, Mike Clark, Peter Huxley, Peter Lewis, Maximillian Birchwood, Melanie Calvert

**Affiliations:** 1 School of Health and Population Sciences, University of Birmingham, Birmingham B15 2TT, UK; 2 The McPin Foundation, London SE1 OEH, UK; 3 Institute of Translational Medicine, University of Liverpool, Liverpool L69 3BX, UK; 4 Centre for Health Economics, University of Manchester, Manchester M13 9PL, UK; 5 Division of Health Research, Lancaster University, Lancaster LA1 4YG, UK; 6 Centre for Clinical Trials and Health Research, Plymouth University, Plymouth PL4 8AA, UK; 7 Institute of Population Health, University of Manchester, Manchester M13 9PL, UK; 8 NIHR School for Social Care Research, London School of Economics and Political Science, London WC2A 2AE, UK; 9 Centre for Mental Health and Society, Bangor University, Bangor LL57 2DG, UK; 10 Birmingham and Solihull Mental Health Foundation Trust, Birmingham B1 3RB, UK; 11 Mental Health and Wellbeing, University of Warwick, Warwick CV4 7AL, UK

## Background

Randomised controlled trials can provide robust evidence to inform clinical care of mental health service users. A core outcome set (COS) for use in research into schizophrenia and bipolar has the potential to reduce reporting bias and increase the ability of reviewers to synthesise results of randomised controlled trials. There is no core outcome set currently available for use in this research area.

The aim of this study is to develop a COS for use in research into schizophrenia and bipolar disorder in a community setting.

## Materials and methods

A group of participants representing the key stakeholder groups, including service users, carers, health and social care professionals and commissioners, will be recruited from the United Kingdom.

Focus groups and one-to-one interviews, led by academic and service user researchers, will seek to identify clinical, social, psychological and physical outcomes that are important to key stakeholders. An iterative, constant comparative and thematic analysis will identify key outcomes and will be supplemented by outcomes identified through a review of literature.

An online, three round, Delphi study with key stakeholders will reduce the range of potential outcomes to a smaller core set. On completion of the Delphi Study a face-to-face consensus meeting will be held to ratify the final outcomes.

A systematic or rapid literature review will assess the properties of existing measures used in research with bipolar and schizophrenia populations. Measures identified will be matched with the outcomes from the Delphi study for consideration and confirmation at a later stakeholder meeting.

## Conclusions

A COS represents the minimum measurement requirement for trials within a research area. It is anticipated that this work will increase the use of stakeholder relevant outcomes and improve our ability interpret and compare the results of studies involving people with schizophrenia and bipolar in a community setting.

*This abstract is dedicated to the memory of Helen Lester*, *Professor of Mental Health at the University of Birmingham UK who led the PARTNERS-2 programme grant development and is sadly missed by colleagues.*

*Funded by an NIHR Programme Grant* (RP-PG-0611-20004)

